# A screening strategy based on machine learning for diagnostic biomarkers in small cell lung cancer

**DOI:** 10.1371/journal.pone.0339195

**Published:** 2026-01-22

**Authors:** Yifeng Pan, Xuansheng Ding, Wenyun Duan, Liangbiao Wang, Yong Dai, Rongrong Han, Shubei Wang, Mingquan Guo

**Affiliations:** 1 School of Medicine, Anhui University of Science and Technology, Huainan, Anhui, China; 2 Key Laboratory of Industrial Dust Deep Reduction and Occupational Health and Safety of Anhui Higher Education Institutes, Huainan, Anhui, China; 3 Anhui Province Engineering Laboratory of Occupational Health and Safety, Huainan, Anhui, China; 4 School of Basic Medicine and Clinical Pharmacy, China Pharmaceutical University, Nanjing, Jiangsu, China; 5 School of Mechanics and Photoelectric Physics, Anhui University of Science and Technology, Huainan, Anhui, China; Marshall University, Joan C. Edwards School of Medicine, UNITED STATES OF AMERICA

## Abstract

Small cell lung cancer (SCLC) is the most aggressive subtype with high mortality rates due to the lack of specific diagnostic biomarkers to delay the optimal opportunity for treatment. Traditional biomarkers, such as neuron-specific enolase (NSE) or pro-gastrin-releasing peptide (ProGRP), have insufficient specificity and sensitivity to meet the demands of clinical diagnosis. Exosome and its contents have become burgeoning cancer biomarkers due to their diverse molecular cargo to achieve intercellular communication. Herein, a novel machine learning strategy was reported for rapid, efficient screening of biomarkers and identified an optimal exosome RNA combination as diagnostic biomarker of SCLC. Firstly, RNA sequencing data from 111 SCLC patients and 362 healthy controls were obtained from the exoRBase 2.0 and 3.0 databases. The machine learning methods were employed to select specific RNA by using 20 iterations with 10-fold nested cross-validation for SCLC diagnosis. Then, an optimal combination of three exosome RNAs (LINC00989, CXCL5, and MAP3K7CL) was confirmed and achieved excellent diagnostic performance (area under the curve (AUC) of 0.950, sensitivity of 0.936, and specificity of 0.892). Finally, an independent validation cohort containing tissue-based RNA expression data for two biomarkers (CXCL5 and MAP3K7CL) from 79 SCLC patients and 7 standard controls was used to evaluate the diagnostic performance of the selected RNAs. The results demonstrated modest diagnostic performance in tissue samples (AUC = 0.718) with two biomarkers, indicating potential cross-tissue applicability despite the limitations of incomplete biomarker coverage. In addition, a specificity analysis of exosome RNA data, including gastric cancer, hepatocellular carcinoma, and breast cancer, demonstrated significant specificity for SCLC. Therefore, the novel biomarker screening strategy integrating nested cross-validation with multiple machine learning algorithms successfully established to offer a potentially valuable protocol for early SCLC diagnosis and other cancers.

## 1. Introduction

Small cell lung cancer (SCLC) with extremely high mortality rates is a highly aggressive subtype of lung cancer due to distinctive features including rapid proliferation, high propensity for early metastasis, and poor prognosis [1–3]. The five-year survival rate for SCLC patients remains consistently low at 14–15%, with early-stage SCLC patients having a median survival of less than 2 years, while metastatic patients survive approximately 1 year. Due to the lack of specific biomarkers for early diagnosis, most SCLC patients are confirmed at an extensive stage [4,5], which delays the optimal opportunity for treatment. Thus, screening of early-stage SCLC is crucial for reducing patient mortality.

Currently, SCLC determination methods primarily include three categories: medical imaging examinations, histopathological biopsy, and liquid biopsy. Although low-dose spiral computed tomography (CT) screening represents the standard method for early lung cancer screening and diagnosis, this approach suffers from high false-positive rates and requires multiple consecutive monitoring sessions, resulting in excessive radiation exposure for patients [6,7]. Histopathological biopsy, as the gold standard of diagnosis for lung cancer, requires obtaining surgical tissue sections for lesion tissues, which causes substantial physical harm to patients and is unsuitable for early screening and diagnosis. Liquid biopsy is a non-invasive detection method for early cancer through analysis of tumor biomarkers in body fluids, including blood, saliva, and urine. These strategies are characterized by a non-invasive nature, minimal patient harm, and the capability for continuous monitoring of high-risk patients. Despite the clinical application of traditional biomarkers such as neuron-specific enolase (NSE) and pro-gastrin-releasing peptide (ProGRP) in SCLC diagnosis, their specificity and sensitivity remain insufficient to meet clinical demands [8,9]. Therefore, the discovery of highly sensitive and specific diagnostic biomarkers and the establishment of high-accuracy early diagnostic methods hold significant practical implications for improving cancer patient survival rates.

Exosomes are membrane-bound vesicular structures ranging from 40–160 nm that are secreted by cells and contain various biological information molecules, including proteins, nucleic acids, and lipids. Exosomes are widely distributed in bodily fluids such as blood and saliva [10], which enter circulatory fluids through exocytosis from cells and are subsequently uptaken by recipient cells, facilitating intercellular information transfer and closely participating in all stages of tumor development and progression [11,12]. Therefore, exosomes can better preserve cancer cell-related biological information, making them excellent potential biomarkers for lung cancer diagnosis. However, research on the correlation between exosomes and their contents with lung cancer, as well as pathway studies, remains limited, which severely restricts the research and application of exosomes as lung cancer biomarkers in early diagnosis, precision treatment, and prognosis. With the rapid development of high-throughput sequencing and machine learning technologies, bioinformatics research has become an important tool for biomarker screening in small-sample and high-dimensional data. Compared to traditional differential expression analysis, machine learning methods can not only capture complex gene interactions but also effectively avoid overfitting issues through cross-validation and feature stability assessment, significantly improving the reliability and generalization capability of biomarkers [13,14]. Therefore, machine learning-based biomarker screening strategies coupled with exosome sequencing data provide new research directions for early diagnosis of malignant tumors such as lung cancer.

In this work, we proposed a novel machine learning strategy for rapid and efficient screening of lung cancer biomarkers and pathway analysis. Firstly, we systematically analyzed exosome RNA expression profiles through multiple feature selection strategies and nested cross-validation by utilizing machine learning methods and three exosome RNA biomarkers were successfully screened with potential diagnostic biomarkers (LINC00989, CXCL5, and MAP3K7CL). These biomarkers demonstrated excellent diagnostic performance with an area under the curve (AUC) of 0.950, a sensitivity of 0.936, and a specificity of 0.892 by receiver operating characteristic (ROC) analysis. Furthermore, we validated the three candidate RNA biomarkers using SCLC tissue and other cancer exosome RNA expression profile datasets, demonstrating that the selected RNA biomarkers possess high specificity, sensitivity, and good generalization capability. Therefore, we successfully established a novel biomarker screening strategy for large-sample and high-dimensional blood exosome RNA data, which offered new protocols for early SCLC diagnosis. This work not only expands the range of SCLC diagnostic biomarkers but also identifies potential new targets for SCLC, with significant research implications for reducing SCLC patient mortality.

## 2. Methods

### 2.1 Acquisition of exosome RNA expression data

Training Dataset Acquisition: Blood exosome RNA expression profile data were obtained from the exoRBase 2.0 (http://exorbase.org:8080) and 3.0 (http://www.exorbase.org/) databases for SCLC diagnostic biomarker screening and model construction. Data from exoRBase 2.0 were accessed on 19 February 2025, and data from exoRBase 3.0 were accessed on 1 November 2025 for research purposes. The combined dataset comprised exosome RNA transcriptome sequencing data from 111 SCLC patients and 362 healthy controls, encompassing 114,601 RNA features, including 35,517 mRNA/lncRNA and 79,084 circRNA transcripts. The expression matrix was provided in normalized count format, suitable for subsequent differential expression analysis and machine learning modeling.

**Fig 1:**
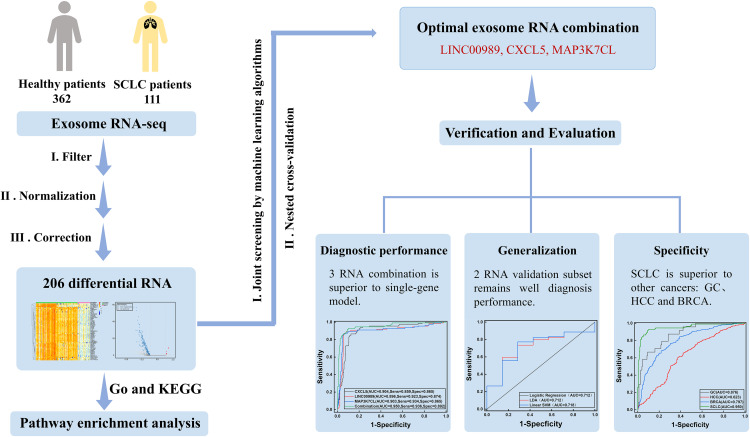
Machine learning-based screening strategy for biomarkers of SCLC.

Validation Dataset Acquisition: To evaluate the generalization capability and cancer specificity of the screened diagnostic biomarkers, validation datasets were obtained from two sources. First, SCLC patient tumor tissue RNA expression profile data were extracted from the Gene Expression Omnibus (GEO) database (https://www.ncbi.nlm.nih.gov/geo/, GSE60052) on 30 March 2025. This cohort included 7 normal lung tissue control samples and 79 SCLC tumor tissue samples for external independent validation. Second, blood exosome RNA expression data from other cancer types were obtained from the exoRBase 2.0 and 3.0 databases, including 24 gastric cancer patients, 417 hepatocellular carcinoma patients, and 382 breast cancer patients, to assess the diagnostic specificity of the screened biomarkers for SCLC. The overall workflow of our machine learning-based biomarker screening strategy is illustrated in Fig 1, which outlines the key steps from data acquisition through validation.

Ethical Considerations: All datasets utilized in this study contain only de-identified, anonymized data obtained from publicly available databases. The authors had no access to information that could identify individual participants during or after data collection. The original studies contributing data to these databases obtained appropriate ethical approvals from their respective institutions. This secondary analysis of publicly available datasets did not require additional ethical approval.

### 2.2 Data preprocessing and differential expression analysis

Initially, technical quality filtering was performed on the acquired data according to literature-reported methods, retaining RNAs that were expressed (non-zero) in at least 80% of samples and had reliable expression levels (counts ≥5) in at least 10% of samples [15]. Log2(CPM + 1) normalization was applied to retained features, with library size differences corrected using the edgeR package. Simultaneously, the ComBat method was employed to eliminate batch effects introduced by RNA types (mRNA/lncRNA and circRNA). Subsequently, DESeq2 was used for exosome RNA differential expression analysis, constructing a statistical model incorporating sample type (SCLC vs. Normal) and RNA type batch effects. Expression differences were assessed through Wald tests, with multiple screening criteria applied (adjusted p-value <0.05, |log2 fold change| > 1.2, mean expression abundance >50) to identify significantly differentially expressed RNAs, providing a candidate set for subsequent feature selection.

### 2.3 Machine learning screening and model evaluation of exosome RNA diagnostic biomarkers

To address the challenges of class imbalance and ensure model robustness, a comprehensive feature screening and model evaluation pipeline based on nested cross-validation was constructed [16,17]. This pipeline integrated three complementary feature selection methods and ensured biomarker reliability through stability assessment [14,18]. Within this framework, three complementary feature selection methods were implemented: (1) LASSO regression, which automatically selects optimal penalty parameters and extracts non-zero coefficient features; (2) Random Forest feature importance analysis, which evaluates feature contributions based on mean impurity reduction; and (3) SVM-RFE recursive feature elimination, which iteratively assesses feature importance. Feature stability scores were calculated through 20 iterations, and cross-validation consistency was evaluated through 10-fold nested cross-validation. Features demonstrating high consistency across cross-validation were selected as candidate biomarkers. Additionally, we systematically evaluated different feature number combinations (2–10 features) to determine the optimal feature subset producing the best diagnostic performance. Throughout the evaluation process, outer 10-fold cross-validation was used to assess model generalization capability, while inner 5-fold cross-validation was employed for feature selection and parameter optimization. The outer fold divided data into training sets (90%) and test sets (10%), ensuring that test set information did not leak into the feature selection process, thereby avoiding performance evaluation bias. We employed Support Vector Machine (SVM) with radial basis function (RBF) kernel as the classifier, optimizing model parameters through grid search. Simultaneously, the Synthetic Minority Oversampling Technique (SMOTE) was used to address class imbalance, thereby improving the model’s sensitivity to SCLC samples. Final model performance was comprehensively evaluated through the ROC curve, AUC, sensitivity, specificity, and accuracy. To assess the reliability of performance estimates, 95% confidence intervals for AUC were calculated based on the distribution of AUC values across the 10 outer folds of nested cross-validation, using the t-distribution to account for the limited number of folds. Model calibration was evaluated using the Hosmer-Lemeshow goodness-of-fit test, and calibration curves were generated to visualize agreement between predicted probabilities and observed outcomes.

### 2.4 Functional enrichment analysis

Gene functional annotation and pathway enrichment analysis were performed using DAVID (Database for Annotation, Visualization and Integrated Discovery) [19] and KOBAS (KEGG Orthology Based Annotation System) [20] online analysis platforms. Gene Ontology (GO) analysis was employed to explore functional enrichment of differentially expressed RNAs in biological processes (BP), molecular functions (MF), and cellular components (CC). Pathway enrichment analysis was conducted through the KEGG (Kyoto Encyclopedia of Genes and Genomes) [21] database to identify molecular pathways associated with SCLC. Hypergeometric tests were used to assess enrichment significance (p-value <0.05, Benjamini-Hochberg correction).

## 3. Results

### 3.1 Differential expression biological functional enrichment analysis of exosome RNAs

In this study, blood exosome RNA sequencing data from 111 SCLC patients and 362 healthy controls were acquired and subjected to dual-layer filtering analysis, retaining 13,188 high-quality RNA features for subsequent analysis. Differential expression analysis was performed using DESeq2, with RNA type incorporated as a covariate to control batch effects. Through stringent screening criteria, 206 significantly differentially expressed RNAs were identified from the 13,188 RNA features.

**Fig 2:**
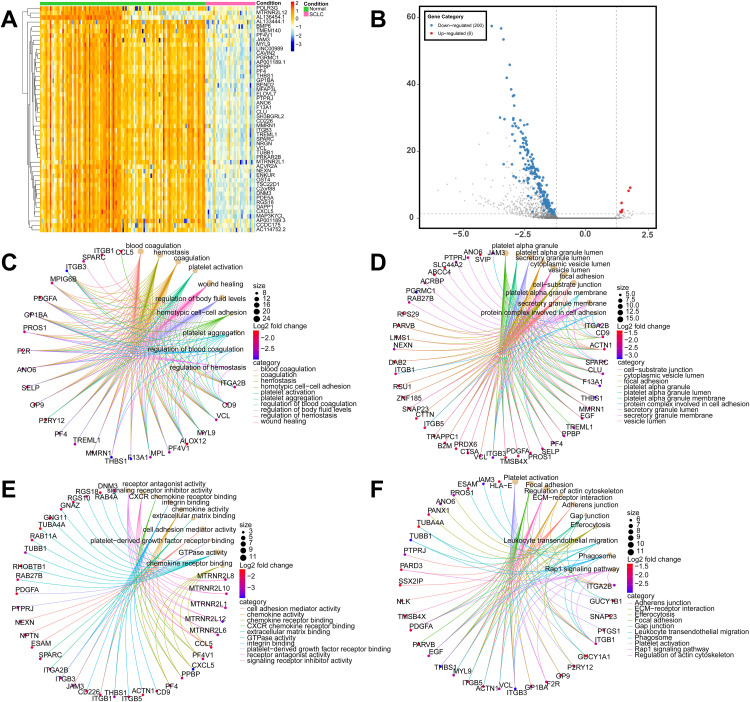
(A) Hierarchical clustering heatmap analysis of the top 50 differentially expressed RNAs. (B) Volcano plot of differential expression of exosome RNAs between SCLC patients and healthy individuals. Red dots represent significantly upregulated genes, blue dots represent significantly downregulated genes, and gray dots represent non-significantly differentially expressed genes. Screening criteria: |log2FC| > 1.2, adjusted p-value <0.05, mean expression >50. Functional enrichment analysis showing the top 10 most significantly enriched terms (ranked by adjusted p-value) for (C) GO biological process analysis, (D) cellular component analysis, and (E) molecular function analysis. (F) KEGG pathway enrichment analysis.

As shown in the hierarchical clustering heatmap (Fig 2A), analysis of the top 50 differentially expressed RNAs ranked by p-value demonstrated that SCLC patients and healthy controls exhibited distinct expression patterns, with relatively consistent expression patterns within the patient group. The volcano plot (Fig 2B) further illustrates the results of differential expression analysis, revealing clear differences in expression levels between SCLC patients and healthy controls. To reveal the potential biological functions of differentially expressed RNAs, GO and KEGG pathway enrichment analyses were performed. Fig 2C-2F displays the top 10 most significantly enriched terms in each category, ranked by adjusted p-value. As shown in Fig 2, differentially expressed RNAs were primarily enriched in biological processes such as blood coagulation and platelet activation (Fig 2C), localized to cellular components including platelet alpha granules (Fig 2D), and participated in molecular functions such as CXCR chemokine receptor binding (Fig 2E). KEGG pathway analysis revealed that these RNAs primarily participated in pathways including platelet activation, focal adhesion, and cytokine-cytokine receptor interaction (Fig 2F). Comprehensive analysis indicated that differentially expressed RNAs in exosomes from SCLC patients were mainly associated with processes related to platelet function, cell adhesion and migration, and immune regulation (S1 Text, S1 Fig), providing important biological foundations for subsequent biomarker screening.

### 3.2 Machine learning screening for optimal SCLC diagnostic biomarker combinations

**Fig 3:**
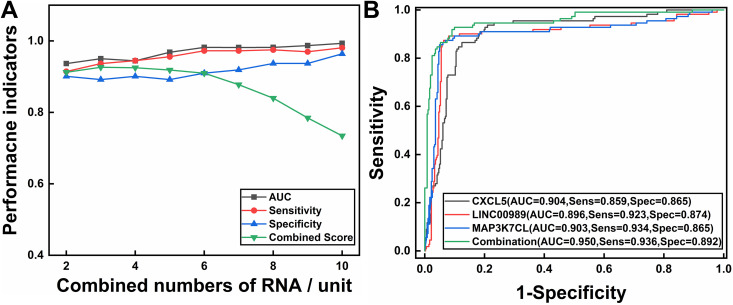
(A) Comparison of diagnostic performance across different feature number combinations. Shows trends in AUC, sensitivity, specificity, and comprehensive scores for 2-10 feature combinations. (B) ROC curve comparison between the 3-exosome RNA combination and individual exosome RNA models.

To further screen exosome RNA biomarkers associated with small cell lung cancer, three complementary feature selection algorithms (LASSO regression, Random Forest feature importance analysis, and SVM-RFE) were applied to the 206 differentially expressed RNAs. Through 20 iterations to calculate stability scores for each feature and 10-fold nested cross-validation to assess cross-validation consistency, multiple high-stability candidate features were identified (S1 Table), ultimately determining the optimal biomarker combination consisting of LINC00989, CXCL5, and MAP3K7CL (Table 1). These three exosome RNAs were identified as important features across all algorithms, demonstrating the highest feature selection consistency (10/10) and ranking highest in p-value significance, all being significantly downregulated in exosomes from SCLC patients, indicating their high reliability and stability as SCLC diagnostic biomarkers. As shown in Fig 3A, the combination of three features demonstrated the best comprehensive score, achieving excellent diagnostic performance while maintaining a relatively small number of features. ROC curve analysis results (Fig 3B) showed that although individual exosome RNAs all demonstrated good diagnostic capability (AUC all equal to 0.89), the 3-exosome RNA combinations exhibited superior comprehensive diagnostic performance (AUC = 0.950, 95% CI: 0.910–0.985; sensitivity = 0.936; specificity = 0.892).

**Table 1:** Feature Selection Screening Process and Stability Assessment Results.

RNA	Feature Selection Consistency Score	Log2FC	Adjusted p-value
CXCL5	10/10	−3.80	3.22e-58 (*p* < 0.05)
LINC00989	10/10	−3.43	1.65e-57 (*p* < 0.05)
MAP3K7CL	10/10	−3.33	3.28e-54 (*p* < 0.05)

Model calibration was evaluated using the Hosmer-Lemeshow goodness-of-fit test to assess the agreement between predicted probabilities and observed outcomes. As shown in S3 Fig, the calibration curve of 3-exosome RNA combination (CXCL5, LINC00989, MAP3K7CL) aligns well with the ideal calibration line (calibration slope = 0.933, Brier score = 0.060, p = 0.795), particularly in the high-probability range (>0.7) and most relevant for clinical diagnosis, which indicated excellent agreement between predicted probabilities and observed frequencies. The calibration slope close to 1.0 indicates accurate probability predictions, while the low Brier score confirms high predictive accuracy. These results indicate that the 3-exosome RNA combination provides not only effective discrimination between SCLC and normal cases, but also accurate probability estimates for clinical risk assessment. Therefore, the advantage of the multi-biomarker combination lies in its ability to integrate complementary information from different molecular markers, providing a more comprehensive of disease characteristics and achieving a more accurate diagnosis.

### 3.3 Comparative analysis of the biomarker screening process

**Fig 4:**
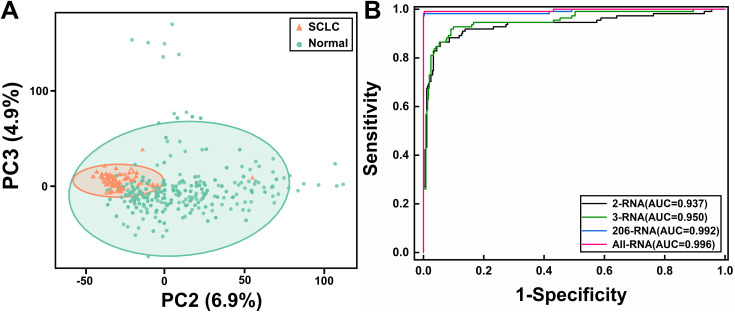
(A) Principal component analysis based on 13,188 RNA features. Orange triangles represent SCLC patients, green circles represent healthy controls. (B) ROC curve comparison of four models: All RNA features, 206 differentially expressed RNAs, 3-exosome RNA combination models, and 2-exosome RNA combination models.

To investigate differences in exosome RNA expression between SCLC patients and healthy individuals, principal component analysis (PCA) was first applied to the 13,188 preprocessed RNA features to extract principal components for unsupervised clustering. As shown in Fig 4A, SCLC patient samples (orange triangles) and healthy control samples (green circles) presented as two distinct clusters in the principal component space, with the two groups forming relatively independent clustering regions, indicating that exosome RNAs can effectively distinguish SCLC patients from healthy individuals.

To systematically evaluate the rationality of each step in the screening process, we compared the diagnostic performance of four models: a full-feature diagnostic model based on 13,188 preprocessed RNAs, a diagnostic model based on 206 differentially expressed RNAs after initial screening, our final screened 3-exosome RNA combination (LINC00989, CXCL5, and MAP3K7CL) diagnostic model, and a 2-exosome RNA model (CXCL5 and MAP3K7CL) for comparison with the validation dataset. As shown in Fig 4B, ROC curve analysis results demonstrated that the model based on 13,188 RNAs exhibited excellent diagnostic efficacy (AUC = 0.996, red curve). The 206 differentially expressed RNA combination showed similarly excellent performance (AUC = 0.992, blue curve), as removing substantial interfering information while retaining key features maintained diagnostic performance. The final screened 3-exosome RNA combination maintained high diagnostic accuracy (AUC = 0.950, green curve) while dramatically reducing the number of features (98.5% reduction compared to 206 differentially expressed RNAs), with only a 4.2% decrease compared to the 206 differentially expressed RNA model. This result indicates that the feature selection process effectively extracted the most diagnostically valuable information from the 206 differentially expressed RNAs. The 2-exosome RNA combination (CXCL5 and MAP3K7CL, AUC = 0.937, black curve) is only marginally lower than the 3-exosome RNA combination.

### 3.4 Validation and analysis of SCLC diagnostic biomarkers in external datasets

To evaluate the generalization capability of the constructed diagnostic model, SCLC-related datasets were searched in the GEO database for validation. Since the GEO dataset contained expression data for only one mRNA (CXCL5 and MAP3K7CL), lacking data for LINC00989, the diagnostic performance of the two-exosome mRNA combination was evaluated. Due to the limited sample size in the validation cohort (79 SCLC patients and 7 controls), we employed linear classification models to avoid overfitting. Among the tested models, Linear SVM achieved optimal performance with an AUC value of 0.718 (95% CI: 0.535–0.901), sensitivity of 0.714, and specificity of 0.696 (Table 2, S2 Fig). Logistic Regression (LR) and Linear Discriminant Analysis (LDA) demonstrated comparable performance (AUC = 0.712, 95% CI: 0.534–0.890). Despite the absence of LINC00989 expression data and the sample type mismatch (tissue vs. exosome), the two available mRNA biomarkers still achieved clinically meaningful diagnostic performance, indirectly validating the effectiveness of our feature selection method.

**Table 2:** Performance of Two Exosome RNA (CXCL5, MAP3K7CL) Combination in External Validation Set.

Model	AUC (95% CI)	Sensitivity	Specificity
Linear SVM	0.718 (0.535-0.901)	0.714	0.696
LR	0.712 (0.534-0.890)	0.714	0.696
LDA	0.712 (0.534-0.890)	0.714	0.696

**Fig 5:**
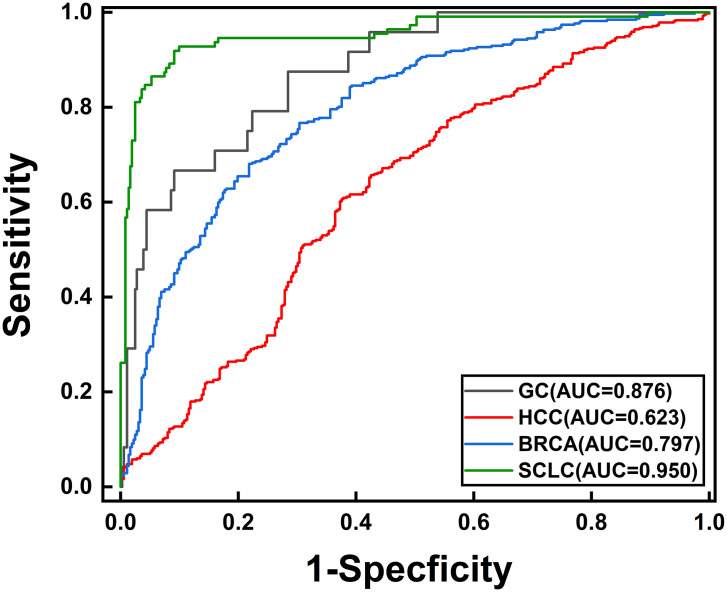
ROC curves of the 3-exosome RNA combination across different cancer types. SCLC AUC = 0.950, gastric cancer AUC = 0.876, hepatocellular carcinoma AUC = 0.623, breast cancer AUC = 0.797.

To further validate the diagnostic specificity of the 3-exosome RNA combination for SCLC, exosome RNA expression profiles from three other cancer types were obtained from the same database, including gastric cancer, hepatocellular carcinoma, and breast cancer. As shown in Fig 5, ROC curve analysis results revealed that the 3-exosome RNA combination demonstrated significant performance differences across different types of cancer. In gastric cancer, the 3-exosome RNA combination achieved an AUC value of 0.876, in hepatocellular carcinoma, the AUC value was 0.623, and in breast cancer, the AUC value reached 0.797. However, although the highest diagnostic accuracy was observed in SCLC (AUC = 0.950), gastric cancer and breast cancer demonstrated accurate diagnosis performance (AUC = 0.876). These results suggest that while some biomarkers may share expression patterns across certain cancer types [24,25], the three-biomarker combination achieves optimal diagnostic performance for SCLC, demonstrating its clinical utility as an SCLC-specific diagnostic panel.

## 4. Discussion

Through bioinformatics analysis and rigorous feature selection strategies, this study successfully screened three exosome RNAs (LINC00989, CXCL5, and MAP3K7CL) from blood exosome RNA expression profiles of SCLC patients as potential diagnostic biomarkers for SCLC. The screened three exosome RNAs and their combination demonstrated excellent diagnostic performance in distinguishing SCLC patients from healthy individuals, with an AUC value of 0.950, sensitivity of 0.936, and specificity of 0.892, providing new biomarkers for accurate SCLC diagnosis while offering new perspectives for investigating the molecular pathological mechanisms of SCLC. The identification of these biomarkers was achieved through integration of three complementary feature selection algorithms (LASSO, Random Forest, and SVM-RFE) with stability assessment across 20 iterations and nested cross-validation, addressing the challenges of small-sample, high-dimensional data analysis and enhancing the reliability and robustness of biomarker discovery in SCLC.

The diagnostic challenges in SCLC are primarily manifested in two aspects: first, most patients are already in advanced stages at diagnosis, with traditional serological markers NSE and ProGRP having limited diagnostic performance; second, the high molecular heterogeneity of SCLC makes early diagnosis difficult to achieve with single biomarkers. Compared to these traditional protein biomarkers, our 3-RNA panel demonstrates several advantages. Traditional markers are released through cell lysis and require substantial tumor burden, while exosome RNAs are actively secreted and directly reflect cellular transcriptional states, potentially enabling earlier detection [8,9]. Our 3-RNA combination achieved substantially higher diagnostic performance (sensitivity 93.6%, specificity 89.2%). Additionally, the multi-biomarker combination captures multiple biological dimensions, including immune regulation (CXCL5), inflammatory signaling (MAP3K7CL), and systemic platelet-related responses (LINC00989), providing more comprehensive disease characterization than traditional protein markers. In this context, the multi-biomarker combination strategy of exosome RNAs demonstrates unique advantages. The 3-exosome RNA combination screened through machine learning algorithms can integrate complementary molecular information, effectively overcome the limitations of single biomarkers, and provide a new technical pathway for precise SCLC diagnosis and high-risk population screening.

Compared to existing research, this study possesses several distinctive features: the 3-exosome RNA combination shows higher sensitivity and specificity than traditional SCLC serological markers (sensitivity typically 60–70%), indicating that exosome RNAs are excellent diagnostic biomarkers, providing new possibilities for early clinical screening of lung cancer patients. The application of nested cross-validation and SMOTE techniques effectively addressed the challenges of class imbalance, ensuring the reliability of model evaluation.

Based on the GEO dataset, we evaluated the diagnostic performance of two exosome mRNAs (CXCL5 and MAP3K7CL). Despite lacking LINC00989 data and the fundamental difference between tissue and exosome samples, the combination of two exosome mRNAs achieved clinically meaningful diagnostic efficacy in the independent validation cohort (Linear SVM model AUC = 0.718), considering the constraints of incomplete biomarker coverage and limited control sample size (n = 7). This result indirectly validates the effectiveness of our feature selection method, though future validation using exosome-based SCLC datasets with complete biomarker coverage would provide more substantial evidence for clinical translation. Furthermore, specificity analysis using exosome RNA data from other cancers revealed that the 3-exosome RNA combination demonstrated variable performance across cancer types (gastric cancer, AUC = 0.876; hepatocellular carcinoma, AUC = 0.623; breast cancer, AUC = 0.797), with SCLC showing the highest diagnostic accuracy (AUC = 0.950). While the model shows cross-reactivity with specific cancer types, particularly gastric cancer, it achieves optimal performance in SCLC, supporting its potential clinical utility for SCLC diagnosis.

The three screened biomarkers (LINC00989, CXCL5, and MAP3K7CL) were all significantly downregulated in SCLC exosomes, possessing crucial biological significance. LINC00989 is a long non-coding RNA that functions as a tumor suppressor in various cancers, primarily participating in tumor progression through regulation of platelet function and immune responses [22–25]. CXCL5, as a key chemokine, regulates immune responses and cell migration through CXCR2 receptors, and its downregulation in exosomes may reflect the molecular mechanisms by which SCLC cells maintain an immunosuppressive state [24,26–29]. MAP3K7CL, as a negative regulator of the p38 MAPK signaling pathway, leads to excessive activation of pro-inflammatory pathways when downregulated, while affecting B cell and T cell functional regulation, participating in the formation of immune escape mechanisms [30–35]. The analytical results of this study are highly consistent with reported findings, indicating that the screened exosome RNAs play important regulatory roles in processes such as platelet function, immune regulation, and inflammatory signaling in SCLC (S2 Text).

Our 3-exosome RNA combination has vast potential for clinical translation, which covers three key aspects: technical feasibility, clinical workflow integration, and practical advantages. First, the biomarker panel is readily implementable using existing clinical infrastructure in technical feasibility perspective. Exosome isolation can be performed using commercially available kits with standardized protocols [36], followed by biomarker measurement using standard RT-qPCR technology widely available in clinical laboratories [37], which ensured reproducibility across different centers and reducing technical barriers to adoption. Second, regarding clinical workflow integration, we propose a tiered diagnostic strategy. The 3-exosome RNA combination could serve as initial screening for high-risk populations (heavy smokers aged >50 years) [38], with positive results triggering confirmatory testing with traditional biomarkers and imaging, while tissue biopsy would be reserved for concordant positive cases. This stepwise approach allows seamless integration into existing lung cancer screening programs without requiring major modifications to established clinical pathways. Third, this strategy offers significant advantages in both cost-effectiveness and patient care in practical implementation standpoint. By improving patient triage and risk stratification, the approach has the potential to reduce unnecessary invasive procedures and associated diagnostic costs [39]. The blood-based test provides additional benefits, including non-invasive sampling, suitability for repeated monitoring, and absence of radiation exposure [40].

Clinical translation of these findings requires several additional efforts in three key directions. First, validation, using independent exosome-based SCLC cohorts with complete biomarker coverage (including LINC00989), is needed to address the current tissue-based validation limitations. Second, multi-center studies across diverse populations and technical platforms should be established to achieve good reproducibility and optimal decision thresholds. Third, longitudinal studies tracking treatment response and recurrence detection could expand the clinical utility of this biomarker panel. These studies will facilitate clinical translation of the 3-exosome RNA combination for early SCLC detection.

## 5. Conclusions

In this work, a novel biomarker screening strategy coupled with bioinformatics analysis and machine learning methods, suitable for large sample, high-dimensional data, was developed by combining multi-algorithm fusion feature selection with nested cross-validation. 3-exosome RNA diagnostic biomarkers (LINC00989, CXCL5, and MAP3K7CL) were successfully screened from blood exosome RNA expression profiles of SCLC patients. The screened three exosome RNAs and their combination demonstrated excellent performance in diagnosing SCLC patients versus healthy individuals (AUC = 0.950, sensitivity = 0.936, specificity = 0.892). In addition, validation analysis using exosome RNA expression data of other cancers revealed that the 3-exosome RNA combination achieved optimal diagnostic performance for SCLC, demonstrating 3-exosome RNA combination was the most excellent diagnostic panel for SCLC in potential clinical utility. This provides new research protocols for SCLC-specific biomarker screening and early diagnosis, holding significant research importance in precision lung cancer diagnosis.

## Supporting information

S1 Text Functional enrichment analysis of differentially expressed RNAs in SCLC exosomes. (DOCX)

S2 Text Molecular mechanism analysis of SCLC exosome RNA diagnostic biomarkers. (DOCX)

S1 Table Detailed evaluation results of all high-stability RNAs in the feature selection process. (DOCX)

S1 Fig GO and KEGG pathway enrichment analysis of differentially expressed RNAs. (DOCX)

S2 Fig ROC curves for external validation using different machine learning algorithms. (DOCX)

S3 Fig Calibration curve for the 3-exosome RNA combination. (DOCX)

## References

[1] de Martel C, Georges D, Bray F, Ferlay J, Clifford GM. Global burden of cancer attributable to infections in 2018: a worldwide incidence analysis. Lancet Glob Health. 2020;8(2):e180–90. doi: 10.1016/S2214-109X(19)30488-7 31862245

[2] Siegel RL, Miller KD, Wagle NS, Jemal A. Cancer statistics, 2023. CA Cancer J Clin. 2023;73(1):17–48. doi: 10.3322/caac.21763 36633525

[3] Liu M, Hu S, Yan N, Popowski KD, Cheng K. Inhalable extracellular vesicle delivery of IL-12 mRNA to treat lung cancer and promote systemic immunity. Nat Nanotechnol. 2024;19(4):565–75. doi: 10.1038/s41565-023-01580-3 38212521 PMC12930425

[4] Siegel RL, Miller KD, Fuchs HE, Jemal A. Cancer Statistics, 2021. CA Cancer J Clin. 2021;71(1):7–33. doi: 10.3322/caac.21654 33433946

[5] Sabari JK, Lok BH, Laird JH, Poirier JT, Rudin CM. Unravelling the biology of SCLC: implications for therapy. Nat Rev Clin Oncol. 2017;14(9):549–61. doi: 10.1038/nrclinonc.2017.71 28534531 PMC5843484

[6] de Goede OM, Nachun DC, Ferraro NM, Gloudemans MJ, Rao AS, Smail C, et al. Population-scale tissue transcriptomics maps long non-coding RNAs to complex disease. Cell. 2021;184(10):2633-2648.e19. doi: 10.1016/j.cell.2021.03.050 33864768 PMC8651477

[7] Burke M, Rashdan S. Management of Immune-Related Adverse Events in Patients With Non-Small Cell Lung Cancer. Front Oncol. 2021;11:720759. doi: 10.3389/fonc.2021.720759 34660286 PMC8514873

[8] Huang Z, Xu D, Zhang F, Ying Y, Song L. Pro-gastrin-releasing peptide and neuron-specific enolase: useful predictors of response to chemotherapy and survival in patients with small cell lung cancer. Clin Transl Oncol. 2016;18(10):1019–25. doi: 10.1007/s12094-015-1479-4 26886220

[9] Li L, Zhang Q, Wang Y, Xu C. Evaluating the diagnostic and prognostic value of serum TuM2-PK, NSE, and ProGRP in small cell lung cancer. J Clin Lab Anal. 2023;37(7):e24865. doi: 10.1002/jcla.24865 37088873 PMC10220296

[10] Li W, Liu J-B, Hou L-K, Yu F, Zhang J, Wu W, et al. Liquid biopsy in lung cancer: significance in diagnostics, prediction, and treatment monitoring. Mol Cancer. 2022;21(1):25. doi: 10.1186/s12943-022-01505-z 35057806 PMC8772097

[11] Hoshino A, Kim HS, Bojmar L, Gyan KE, Cioffi M, Hernandez J, et al. Extracellular Vesicle and Particle Biomarkers Define Multiple Human Cancers. Cell. 2020;182(4):1044-1061.e18. doi: 10.1016/j.cell.2020.07.009 32795414 PMC7522766

[12] Bao H, Tian Y, Wang H, Ye T, Wang S, Zhao J, et al. Exosome-loaded degradable polymeric microcapsules for the treatment of vitreoretinal diseases. Nat Biomed Eng. 2024;8(11):1436–52. doi: 10.1038/s41551-023-01112-3 37872369

[13] Roberts DR, Bahn V, Ciuti S, Boyce MS, Elith J, Guillera‐Arroita G, et al. Cross‐validation strategies for data with temporal, spatial, hierarchical, or phylogenetic structure. Ecography. 2017;40(8):913–29. doi: 10.1111/ecog.02881

[14] Saeys Y, Inza I, Larrañaga P. A review of feature selection techniques in bioinformatics. Bioinformatics. 2007;23(19):2507–17. doi: 10.1093/bioinformatics/btm344 17720704

[15] Tarazona S, García-Alcalde F, Dopazo J, Ferrer A, Conesa A. Differential expression in RNA-seq: a matter of depth. Genome Res. 2011;21(12):2213–23. doi: 10.1101/gr.124321.111 21903743 PMC3227109

[16] Varma S, Simon R. Bias in error estimation when using cross-validation for model selection. BMC Bioinformatics. 2006;7:91. doi: 10.1186/1471-2105-7-91 16504092 PMC1397873

[17] Krstajic D, Buturovic LJ, Leahy DE, Thomas S. Cross-validation pitfalls when selecting and assessing regression and classification models. J Cheminform. 2014;6(1):10. doi: 10.1186/1758-2946-6-10 24678909 PMC3994246

[18] Haibo He, Garcia EA. Learning from Imbalanced Data. IEEE Trans Knowl Data Eng. 2009;21(9):1263–84. doi: 10.1109/tkde.2008.239

[19] Huang DW, Sherman BT, Lempicki RA. Systematic and integrative analysis of large gene lists using DAVID bioinformatics resources. Nat Protoc. 2009;4(1):44–57. doi: 10.1038/nprot.2008.211 19131956

[20] Xie C, Mao X, Huang J, Ding Y, Wu J, Dong S, et al. KOBAS 2.0: a web server for annotation and identification of enriched pathways and diseases. Nucleic Acids Res. 2011;39(Web Server issue):W316-22. doi: 10.1093/nar/gkr483 21715386 PMC3125809

[21] Kanehisa M, Goto S. KEGG: kyoto encyclopedia of genes and genomes. Nucleic Acids Res. 2000;28(1):27–30. doi: 10.1093/nar/28.1.27 10592173 PMC102409

[22] Fang Y, Xiang L, Chen L-M, Sun W-J, Zhai Y-J, Fan Y-C, et al. TNFRSF12A and a new prognostic model identified from methylation combined with expression profiles to predict overall survival in hepatocellular carcinoma. Transl Cancer Res. 2020;9(9):5493–507. doi: 10.21037/tcr-20-1342 35117914 PMC8797803

[23] Sol N, Wurdinger T. Platelet RNA signatures for the detection of cancer. Cancer Metastasis Rev. 2017;36(2):263–72. doi: 10.1007/s10555-017-9674-0 28681241 PMC5557864

[24] Cho O, Kim D-W, Cheong J-Y. Screening Plasma Exosomal RNAs as Diagnostic Markers for Cervical Cancer: An Analysis of Patients Who Underwent Primary Chemoradiotherapy. Biomolecules. 2021;11(11):1691. doi: 10.3390/biom11111691 34827689 PMC8615616

[25] Zhou W, Pang Y, Yao Y, Qiao H. Development of a Ten-lncRNA Signature Prognostic Model for Breast Cancer Survival: A Study with the TCGA Database. Anal Cell Pathol (Amst). 2020;2020:6827057. doi: 10.1155/2020/6827057 32908814 PMC7450318

[26] Deng J, Jiang R, Meng E, Wu H. CXCL5: A coachman to drive cancer progression. Front Oncol. 2022;12:944494. doi: 10.3389/fonc.2022.944494 35978824 PMC9376318

[27] Hu B, Fan H, Lv X, Chen S, Shao Z. Prognostic significance of CXCL5 expression in cancer patients: a meta-analysis. Cancer Cell Int. 2018;18:68. doi: 10.1186/s12935-018-0562-7 29743818 PMC5930840

[28] Sun D, Tan L, Chen Y, Yuan Q, Jiang K, Liu Y, et al. CXCL5 impedes CD8+ T cell immunity by upregulating PD-L1 expression in lung cancer via PXN/AKT signaling phosphorylation and neutrophil chemotaxis. J Exp Clin Cancer Res. 2024;43(1):202. doi: 10.1186/s13046-024-03122-8 39034411 PMC11264977

[29] Hamilton G, Rath B, Klameth L, Hochmair MJ. Small cell lung cancer: Recruitment of macrophages by circulating tumor cells. Oncoimmunology. 2015;5(3):e1093277. doi: 10.1080/2162402X.2015.1093277 27141354 PMC4839345

[30] Niu L, Guo W, Song X, Song X, Xie L. Tumor-educated leukocytes mRNA as a diagnostic biomarker for non-small cell lung cancer. Thorac Cancer. 2021;12(6):737–45. doi: 10.1111/1759-7714.13833 33474835 PMC7952788

[31] Lin W-D, Liao W-L, Chen W-C, Liu T-Y, Chen Y-C, Tsai F-J. Genome-wide association study identifies novel susceptible loci and evaluation of polygenic risk score for chronic obstructive pulmonary disease in a Taiwanese population. BMC Genomics. 2024;25(1):607. doi: 10.1186/s12864-024-10526-5 38886662 PMC11184693

[32] Kilpinen S, Ojala K, Kallioniemi O. Analysis of kinase gene expression patterns across 5681 human tissue samples reveals functional genomic taxonomy of the kinome. PLoS One. 2010;5(12):e15068. doi: 10.1371/journal.pone.0015068 21151926 PMC2997066

[33] Elwakeel E, Brüggemann M, Wagih J, Lityagina O, Elewa MAF, Han Y, et al. Disruption of Prostaglandin E2 Signaling in Cancer-Associated Fibroblasts Limits Mammary Carcinoma Growth but Promotes Metastasis. Cancer Res. 2022;82(7):1380–95. doi: 10.1158/0008-5472.CAN-21-2116 35105690

[34] Nührenberg TG, Stöckle J, Marini F, Zurek M, Grüning BA, Benes V, et al. Impact of high platelet turnover on the platelet transcriptome: Results from platelet RNA-sequencing in patients with sepsis. PLoS One. 2022;17(1):e0260222. doi: 10.1371/journal.pone.0260222 35085240 PMC8794123

[35] Tao Y, Xing S, Zuo S, Bao P, Jin Y, Li Y, et al. Cell-free multi-omics analysis reveals potential biomarkers in gastrointestinal cancer patients’ blood. Cell Rep Med. 2023;4(11):101281. doi: 10.1016/j.xcrm.2023.101281 37992683 PMC10694666

[36] Dilsiz N. A comprehensive review on recent advances in exosome isolation and characterization: Toward clinical applications. Transl Oncol. 2024;50:102121. doi: 10.1016/j.tranon.2024.102121 39278189 PMC11418158

[37] Ren F, Fei Q, Qiu K, Zhang Y, Zhang H, Sun L. Liquid biopsy techniques and lung cancer: diagnosis, monitoring and evaluation. J Exp Clin Cancer Res. 2024;43(1):96. doi: 10.1186/s13046-024-03026-7 38561776 PMC10985944

[38] de Koning HJ, van der Aalst CM, de Jong PA, Scholten ET, Nackaerts K, Heuvelmans MA, et al. Reduced Lung-Cancer Mortality with Volume CT Screening in a Randomized Trial. N Engl J Med. 2020;382(6):503–13. doi: 10.1056/NEJMoa1911793 31995683

[39] Wan JCM, Sasieni P, Rosenfeld N. Promises and pitfalls of multi-cancer early detection using liquid biopsy tests. Nat Rev Clin Oncol. 2025;22(8):566–80. doi: 10.1038/s41571-025-01033-x 40514453

[40] Ma L, Guo H, Zhao Y, Liu Z, Wang C, Bu J, et al. Liquid biopsy in cancer current: status, challenges and future prospects. Signal Transduct Target Ther. 2024;9(1):336. doi: 10.1038/s41392-024-02021-w 39617822 PMC11609310

