# Supplementary material for: A screening strategy based on machine learning for diagnostic biomarkers in small cell lung cancer
Source: PLoS One. 2026 Jan 22;21(1):e0339195. doi: 10.1371/journal.pone.0339195 (PMC12826499; doi:10.1371/journal.pone.0339195)
Supplement: S1 Text — (DOCX) [file pone.0339195.s001.docx]

**S1. Functional enrichment analysis of differentially expressed RNAs in SCLC exosomes**

**S1.1 Detailed analytical methods**

To gain in-depth understanding of the biological functions of the 206 differentially expressed RNAs, we employed a multi-level functional enrichment analysis strategy. First, Gene Ontology analysis was performed using the DAVID (Database for Annotation, Visualization and Integrated Discovery) online platform, encompassing three dimensions: Biological Process (BP), Cellular Component (CC), and Molecular Function (MF). Subsequently, KEGG pathway enrichment analysis was conducted through the KOBAS (KEGG Orthology Based Annotation System) platform. All enrichment analyses employed hypergeometric tests, with significance criteria of adjusted p-value <0.05 and enrichment factor >1.5, applying the Benjamini-Hochberg method for multiple comparison correction.

**S1.2 GO biological processes**

GO biological process analysis revealed key biological mechanisms involving differentially expressed RNAs in SCLC exosomes. The most significantly enriched processes included blood coagulation, hemostasis, coagulation, wound healing, and platelet activation. The enrichment of these processes carries important pathophysiological significance. Abnormalities in blood coagulation and hemostatic function are closely associated with tumor-related thromboembolic complications, which represent one of the common clinical manifestations in SCLC patients. The enrichment of platelet activation processes is particularly noteworthy, as activated platelets not only promote thrombus formation but also facilitate tumor angiogenesis and distant metastasis through the release of vascular endothelial growth factor (VEGF), platelet-derived growth factor (PDGF), and other factors.

**S1.3 Cellular components**

Cellular component analysis revealed that differentially expressed RNAs were primarily localized to structures including platelet alpha granules, platelet alpha granule lumen, and secretory granule lumen. Additionally, significant enrichment was observed in focal adhesion structures. Platelet alpha granules are the largest and most important secretory granules in platelets, containing abundant proteins including coagulation factors, growth factors, chemokines, and angiogenesis regulatory factors. These granules release their contents to the extracellular environment upon platelet activation, participating in hemostasis, inflammatory responses, and tissue repair. In the tumor microenvironment, abnormal release from platelet alpha granules may promote tumor cell invasion and metastasis. The enrichment of focal adhesion components further emphasizes the importance of cell-matrix interactions in SCLC progression.

**S1.4 Molecular functions**

Molecular function analysis revealed key molecular interaction mechanisms involving differentially expressed RNAs. Notably, the most significantly enriched functions included receptor antagonist activity and signaling receptor inhibitor activity, suggesting complex regulatory mechanisms in receptor-mediated signaling pathways in SCLC. The enrichment of CXCR chemokine receptor binding suggests the important role of the chemokine system in SCLC development. The chemokine and receptor system not only regulates immune cell recruitment and activation but also directly affects tumor cell proliferation, migration, and invasion. The enrichment of chemokine activity further supports this perspective. The enrichment of extracellular matrix binding function reflects the complexity of interactions between SCLC cells and the matrix microenvironment, which represents a crucial step in tumor invasion and metastasis.

**S1.5 KEGG pathway enrichment analysis**

KEGG pathway analysis provided a systematic view of molecular pathways involving differentially expressed RNAs. Significant enrichment of the focal adhesion pathway reflects the importance of cell-matrix interactions in SCLC. This pathway involves integrin-mediated cell adhesion, regulating cell morphology, migration, and survival. Enrichment of the platelet activation pathway is consistent with our findings from GO analysis, emphasizing the central role of platelet function in SCLC pathological processes. Enrichment of the regulation of actin cytoskeleton pathway suggests the crucial role of cytoskeletal remodeling in SCLC cells' acquisition of motility capabilities. Additionally, ECM-receptor interaction pathway was significantly enriched, highlighting the critical role of extracellular matrix components in modulating tumor cell behavior and microenvironment remodeling.

**S1.6 Association analysis with SCLC pathological features**

The functional enrichment analysis results are highly consistent with the pathological characteristics of SCLC. SCLC is characterized by rapid proliferation and early extensive metastasis, and our findings of abnormalities in platelet activation, cytoskeletal regulation, cell-matrix interactions, and chemokine systems precisely explain the molecular basis of these clinical features. Platelet activation promotes hematogenous dissemination of tumor cells, cytoskeletal remodeling enhances tumor cell motility, focal adhesion and ECM-receptor interactions facilitate invasion and migration, and dysregulation of the chemokine system creates a favorable migratory microenvironment for tumor cells.

**S1.7 Guiding significance for biomarker screening**

Based on the results of functional enrichment analysis, we focused on genes participating in the aforementioned biological processes as potential diagnostic biomarker candidates. This provided a biological foundation for subsequent machine learning feature screening, improving screening efficiency and biological rationality. The three finally screened biomarkers (LINC00989, CXCL5, and MAP3K7CL) are all closely related to these enriched biological functions, validating the effectiveness of the function-oriented screening strategy.
