# Supplementary material for: A screening strategy based on machine learning for diagnostic biomarkers in small cell lung cancer
Source: PLoS One. 2026 Jan 22;21(1):e0339195. doi: 10.1371/journal.pone.0339195 (PMC12826499; doi:10.1371/journal.pone.0339195)
Supplement: S2 Text — (DOCX) [file pone.0339195.s002.docx]

**S2 Molecular mechanism analysis of SCLC exosome RNA diagnostic biomarkers**

**S2.1 Molecular mechanism and functional analysis of LINC00989**

LINC00989 is a long non-coding RNA that is significantly downregulated in SCLC exosomes (log2FC=-3.43). Research demonstrates that in hepatocellular carcinoma, this lncRNA exhibits a downregulation trend as a component of methylation-driven prognostic models and is significantly associated with patient survival ^[1]^. Tumor-educated platelets (TEPs) studies (encompassing 55 healthy controls and 228 cancer patients) found that LINC00989 is widely downregulated in platelets of cancer patients ^[2]^. These studies support our SCLC exosome data. Related mechanistic studies indicate that LINC00989 is coordinately downregulated with RGS18 (a G-protein receptor negative regulator that inhibits platelet activation) in cervical cancer exosomes, potentially promoting tumor progression through platelet activation ^[3]^, which is highly consistent with the "platelet activation pathway" in SCLC GO enrichment analysis. Additionally, breast cancer research shows its involvement in lncRNA signatures associated with immune responses and cytokine pathways, suggesting its potential role in mediating immune evasion ^[4]^. Comprehensively, LINC00989 may function through three pathways in SCLC: coordinately regulating platelet function genes (such as RGS18) to promote early hematogenous metastasis; dysregulating immune pathways to facilitate immune evasion; and serving as an intercellular communication factor to remodel the tumor microenvironment through downregulation. However, its specific molecular mechanisms in SCLC still require in-depth experimental validation.

**S2.2 Chemokine network regulatory mechanism of CXCL5**

CXCL5 (C-X-C motif chemokine ligand 5) is an important member of the chemokine family that exerts biological functions through binding to CXCR2 receptors, playing a crucial role in inflammatory responses and tumor microenvironment regulation. In our study, CXCL5 showed significant downregulation in SCLC exosomes (log2FC=-3.80), a finding that presents an interesting contrast to its expression patterns in most solid tumor tissues. Extensive research confirms that CXCL5 is significantly upregulated in various malignant tumors including gastric cancer, colorectal cancer, pancreatic cancer, head and neck squamous cell carcinoma, and non-small cell lung cancer, participating in epithelial-mesenchymal transition, tumor cell proliferation, metastatic cell migration, and angiogenesis processes ^[5-6]^. Lung cancer-related research indicates that CXCL5 is significantly overexpressed in cancer cells, leading to upregulated PD-L1 expression through phosphorylation activation of the PXN/AKT signaling pathway, thereby impeding CD8+ T cell immune function and positively correlating with poor patient prognosis. This study also confirmed that CXCL5 can recruit neutrophils, and these PD-L1-positive neutrophils further exacerbate CD8+ T cell exhaustion processes ^[7]^. Importantly, CXCL5 expression patterns in exosomes differ from tissue expression. Cervical cancer plasma exosome studies found that although CXCL5 is upregulated in cervical cancer tissues, it is significantly downregulated in exosomes ^[3]^, highly consistent with our observations in SCLC exosomes. In SCLC-related research, Hamilton et al. found that circulating tumor cells can secrete ENA-78/CXCL5, recruiting neutrophils with angiogenic properties to promote tumor invasiveness and angiogenesis ^[8]^. The downregulation of CXCL5 in exosomes may reflect SCLC cells maintaining an immunosuppressive state by reducing remote transmission of pro-inflammatory signals, which is consistent with the common immunosuppressive phenotype in SCLC patients and directly related to the chemokine activity and extracellular matrix interaction pathways highlighted in our enrichment analysis results.

**S2.3 Signal pathway regulatory function of MAP3K7CL**

MAP3K7CL (MAP3K7 C-terminal like), also known as TAK1L (TGF-beta activated kinase like) or C21orf7, is a kinase-related gene significantly downregulated in SCLC exosomes (log2FC=-3.33). This gene exhibits a consistent downregulation pattern in pulmonary diseases. Niu et al. discovered through RNA sequencing technology analysis that MAP3K7CL is significantly downregulated in tumor-educated leukocytes of non-small cell lung cancer (NSCLC) patients ^[9]^. Lin et al. further identified MAP3K7CL as a disease susceptibility gene in a genome-wide association study of chronic obstructive pulmonary disease in the Taiwanese population ^[10]^. These findings collectively support the important role of MAP3K7CL in pulmonary disease pathogenesis. Large-scale gene expression analysis revealed the unique tissue distribution characteristics of MAP3K7CL. Kilpinen et al.'s systematic analysis of 459 human kinase genes across 5,681 tissue samples found that MAP3K7CL expression has blood/immune tissue specificity (such as lymphoma, peripheral leukocytes), with high expression in lymphoma, mesenchymal stem cells, and peripheral leukocytes, closely associated with multiple biological processes including B cell and T cell signal transduction, immune responses, signal transduction, metabolism, mesoderm development, and cytoskeletal organization ^[11]^. Functional mechanism studies indicate that MAP3K7CL acts as a negative regulator of the p38 MAPK signaling pathway, and its downregulation leads to excessive activation of pro-inflammatory pathways and promotes tumor-associated fibroblast proliferation, clinically associated with prolonged patient survival, establishing its tumor suppressive function ^[12]^. In blood biomarker research, MAP3K7CL is significantly downregulated in platelets of sepsis patients and in cell-free RNA detection of gastrointestinal cancers ^[13-14]^, validating its liquid biopsy value. Based on this research evidence, the significant downregulation of MAP3K7CL in SCLC exosomes has important pathological significance. As a negative regulator of the p38 MAPK signaling pathway, its downregulation will lead to excessive activation of pro-inflammatory pathways, promoting chronic inflammatory states in the tumor microenvironment, which is highly consistent with the inflammation-related pathway alterations found in our enrichment analysis. Additionally, considering the important role of MAP3K7CL in B cell and T cell functional regulation, its downregulation may significantly affect anti-tumor immune responses in SCLC patients, promoting the formation of immune evasion mechanisms.

**References**

1. Fang Y, Xiang L, Chen LM, Sun WJ, Zhai YJ, Fan YC, et al. TNFRSF12A and a new prognostic model identified from methylation combined with expression profiles to predict overall survival in hepatocellular carcinoma. Transl Cancer Res. 2020;9:5493-5506. doi: 10.21037/tcr-20-1342
2. Sol N, Wurdinger T. Platelet RNA signatures for the detection of cancer. Cancer Metastasis Rev. 2017;36:263-272. doi: 10.1007/s10555-017-9674-0
3. Cho O, Kim DW, Cheong JY. Screening plasma exosomal RNAs as diagnostic markers for cervical cancer: an analysis of patients who underwent primary chemoradiotherapy. Biomolecules. 2021;11:1691. doi: 10.3390/biom11111691
4. Zhou W, Pang Y, Yao Y, Qiao H. Development of a ten-lncRNA signature prognostic model for breast cancer survival: a study with the TCGA database. Anal Cell Pathol. 2020;2020:6827057. doi: 10.1155/2020/6827057
5. Deng J, Jiang R, Meng E, Wu H. CXCL5: a coachman to drive cancer progression. Front Oncol. 2022;12:944494. doi: 10.3389/fonc.2022.944494
6. Hu B, Fan H, Lv X, Chen S, Shao Z. Prognostic significance of CXCL5 expression in cancer patients: a meta-analysis. Cancer Cell Int. 2018;18:212. doi: 10.1186/s12935-018-0562-7
7. Sun D, Tan L, Chen Y, Yuan Q, Jiang K, Liu Y, et al. CXCL5 impedes CD8+ T cell immunity by upregulating PD-L1 expression in lung cancer via PXN/AKT signaling phosphorylation and neutrophil chemotaxis. J Exp Clin Cancer Res. 2024;43:202. doi: 10.1186/s13046-024-03122-8
8. Hamilton G, Rath B, Klameth L, Hochmair MJ. Small cell lung cancer: recruitment of macrophages by circulating tumor cells. Oncoimmunology. 2016;5:e1093277. doi: 10.1080/2162402X.2015.1093277
9. Niu L, Guo W, Song X, Song X, Xie L. Tumor-educated leukocytes mRNA as a diagnostic biomarker for non-small cell lung cancer. Thorac Cancer. 2021;12:737-745. doi: 10.1111/1759-7714.13833
10. Lin WD, Liao WL, Chen WC, Liu TY, Chen YC, Tsai FJ, et al. Genome-wide association study identifies novel susceptible loci and evaluation of polygenic risk score for chronic obstructive pulmonary disease in a Taiwanese population. BMC Genomics. 2024;25:607. doi: 10.1186/s12864-024-10526-5
11. Kilpinen S, Ojala K, Kallioniemi O. Analysis of kinase gene expression patterns across 5681 human tissue samples reveals functional genomic taxonomy of the kinome. PLoS One. 2010;5:e15068. doi: 10.1371/journal.pone.0015068
12. Elwakeel E, Brüggemann M, Wagih J, Lityagina O, Elewa MA, Han Y, et al. Disruption of prostaglandin E2 signaling in cancer-associated fibroblasts limits mammary carcinoma growth but promotes metastasis. Cancer Res. 2022;82:1380-1395. doi: 10.1158/0008-5472.CAN-21-2116
13. Nührenberg TG, Stöckle J, Marini F, Zurek M, Grüning BA, Benes V, et al. Impact of high platelet turnover on the platelet transcriptome: results from platelet RNA-sequencing in patients with sepsis. PLoS One. 2022;17:e0260222. doi: 10.1371/journal.pone.0260222
14. Tao Y, Xing S, Zuo S, Bao P, Jin Y, Li Y, et al. Cell-free multi-omics analysis reveals potential biomarkers in gastrointestinal cancer patients' blood. Cell Rep Med. 2023;4:101199. doi: 10.1016/j.xcrm.2023.101281
