# Supplementary material for: A screening strategy based on machine learning for diagnostic biomarkers in small cell lung cancer
Source: PLoS One. 2026 Jan 22;21(1):e0339195. doi: 10.1371/journal.pone.0339195 (PMC12826499; doi:10.1371/journal.pone.0339195)
Supplement: S1 Table — (DOCX) [file pone.0339195.s003.docx]

**Table S1. Detailed evaluation results of all high-stability RNAs in the feature selection process**

| RNA | Feature Selection Consistency Score | Log2FC | BaseMean | Adjusted p-value |
| --- | --- | --- | --- | --- |
| CXCL5 | 10 | -3.802295759 | 494.9506 | 3.22e-58 |
| LINC00989 | 10 | -3.42601273 | 497.835 | 1.65e-57 |
| MAP3K7CL | 10 | -3.331083847 | 965.555 | 3.28e-54 |
| MTRNR2L12 | 10 | -3.466266246 | 9094.711 | 7.70e-31 |
| AL133444.1 | 1 | -3.289402922 | 139.736 | 2.29e-30 |
| MTRNR2L1 | 9 | -2.833885718 | 182.875 | 3.08e-28 |
| AL136454.1 | 9 | -2.976481931 | 55.70598 | 8.52e-26 |
| MTRNR2L8 | 8 | -2.30839636 | 12552.54 | 1.49e-12 |
| HIST1H3B | 10 | 1.762450948 | 700.8871 | 7.44e-10 |
| MTRNR2L10 | 5 | -2.342282033 | 665.8702 | 2.38e-07 |
| VASH1 | 10 | 1.415816182 | 70.2001 | 2.55e-05 |
| HIST1H3C | 10 | 1.437698994 | 573.2254 | 0.003466134 |
| TRAK2 | 10 | 1.370061248 | 1995.517 | 0.011621221 |
