# Supplementary material for: A screening strategy based on machine learning for diagnostic biomarkers in small cell lung cancer
Source: PLoS One. 2026 Jan 22;21(1):e0339195. doi: 10.1371/journal.pone.0339195 (PMC12826499; doi:10.1371/journal.pone.0339195)
Supplement: S1 Fig — (DOCX) [file pone.0339195.s004.docx]

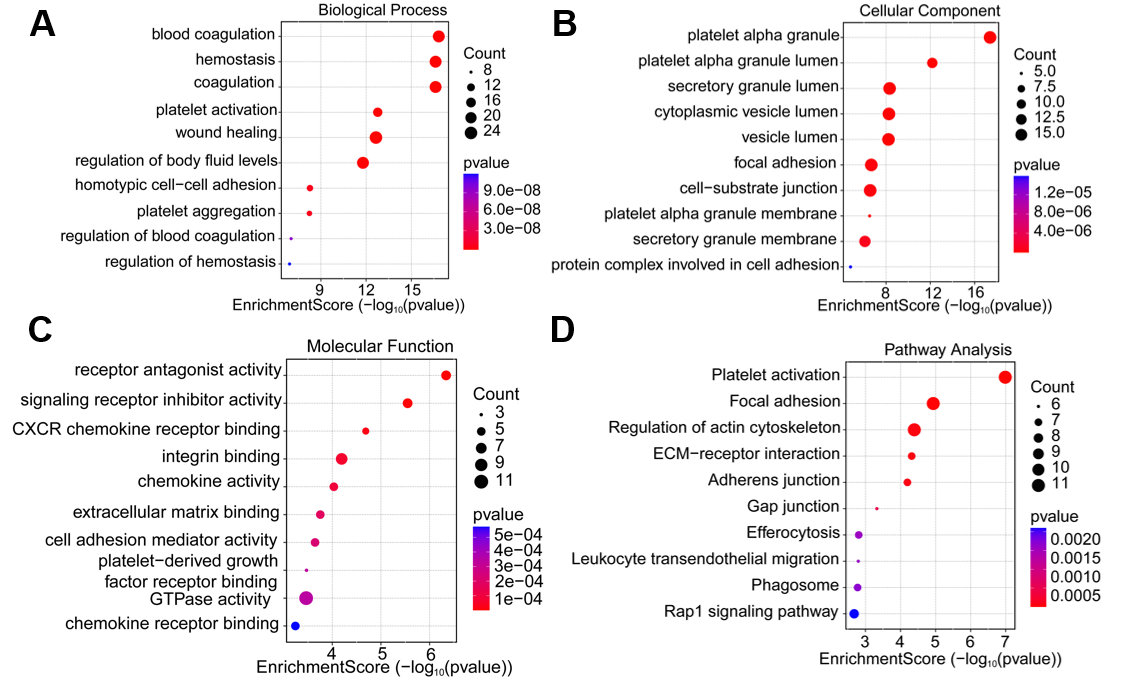


**Fig S1** (A) GO biological process enrichment analysis, (B) GO cellular component enrichment analysis, (C) GO molecular function enrichment analysis, (D) KEGG pathway enrichment analysis. The size of the bubbles indicates the number of enriched genes, and the shade of the color represents the significance level of enrichment (-log_10_ P value).
