# Supplementary material for: A screening strategy based on machine learning for diagnostic biomarkers in small cell lung cancer
Source: PLoS One. 2026 Jan 22;21(1):e0339195. doi: 10.1371/journal.pone.0339195 (PMC12826499; doi:10.1371/journal.pone.0339195)
Supplement: S2 Fig — (DOCX) [file pone.0339195.s005.docx]

**Fig S2** The ROC curves of two exosomal RNA combinations (CXCL5 and MAP3K7CL) in the GEO database (GSE60052) for the diagnosis of SCLC using different machine learning algorithms. Linear SVM achieved the best performance (AUC = 0.718), followed by Logistic Regression (AUC = 0.712) and Linear Discriminant Analysis (LDA, AUC = 0.712).
