# Supplementary material for: A screening strategy based on machine learning for diagnostic biomarkers in small cell lung cancer
Source: PLoS One. 2026 Jan 22;21(1):e0339195. doi: 10.1371/journal.pone.0339195 (PMC12826499; doi:10.1371/journal.pone.0339195)
Supplement: S3 Fig — (DOCX) [file pone.0339195.s006.docx]

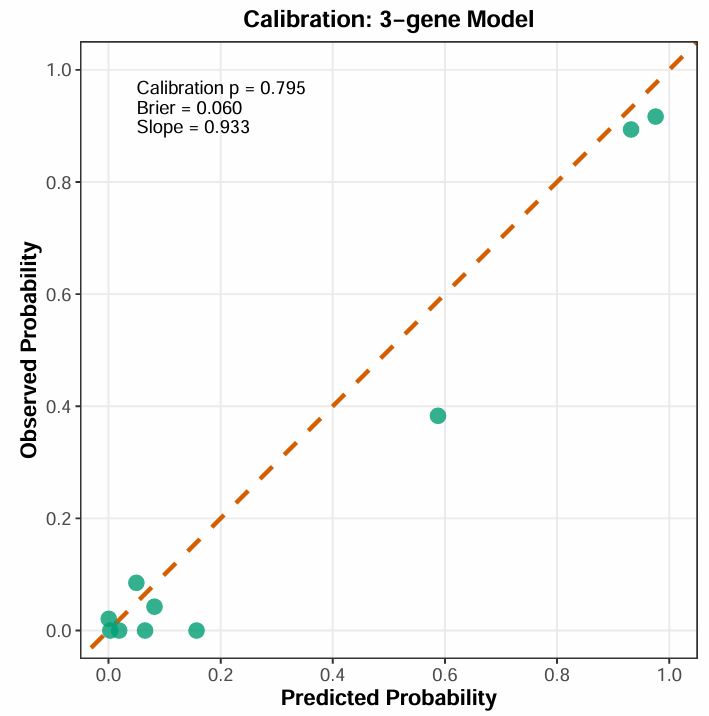


**Fig S3** Calibration curve for the 3-exosome RNA combination (LINC00989, CXCL5, MAP3K7CL). The orange dashed line represents the ideal calibration line. Green dots represent the observed data grouped into deciles. Calibration p = 0.795, Brier score = 0.060, calibration slope = 0.933.
